# Paricalcitol reduces oxidative stress and inflammation in hemodialysis patients

**DOI:** 10.1186/1471-2369-13-159

**Published:** 2012-11-27

**Authors:** María Jesús Izquierdo, Mónica Cavia, Pilar Muñiz, Angel LM de Francisco, Manuel Arias, Javier Santos, Pedro Abaigar

**Affiliations:** 1 Nephrology Service, Complejo Asistencial Universitario de Burgos, C/ Fuenteovejuna 138, Burgos, 09006, Spain; 2 Research Unit. Complejo Asistencial Universitario de Burgos, Burgos, Spain; 3 Área de Bioquímica y Biología molecular de la Universidad de Burgos, Burgos, Spain; 4 Nephrology Service. Hospital Universitario Marques de Valdecilla, Santander, Spain; 5 Unidad de Investigación. Hospital Universitario de Burgos, Burgos. Islas Baleares S/N, 09006, Burgos, Spain; 6 Área de Bioquímica y Biología Molecular, Facultad de Ciencias. Universidad de Burgos, Plaza Misael Bañuelos s/n, Burgos, Spain; 7 Servicio Nefrología. Hospital Universitario Marqués de Valdecilla, Avda. de Valdecilla, s/n, 39008, Santander, Cantabria, Spain; 8 Servicio de Nefrología, Hospital Universitario de Burgos, Islas Baleares S/N, 09006, Burgos, Spain

**Keywords:** Oxidative Stress, Immunomodulation, Receptors, Calcitriol, Paricalcitol, 19-nor-1alpha,25-dihydroxyvitamin D2

## Abstract

**Background:**

Treatment with selective vitamin D receptor activators such as paricalcitol have been shown to exert an anti-inflammatory effect in patients on hemodialysis, in addition to their action on mineral metabolism and independently of parathyroid hormone (PTH) levels. The objective of this study was to evaluate the additional antioxidant capacity of paricalcitol in a clinical setting.

**Methods:**

The study included 19 patients with renal disease on hemodialysis, of whom peripheral blood was obtained for analysis at baseline and three months after starting intravenous paricalcitol treatment. The following oxidizing and inflammatory markers were quantified: malondialdehyde (MDA), nitrites and carbonyl groups, indoleamine 2,3-dioxygenase (IDO), tumor necrosis factor alfa (TNF-α), interleukin-6 (IL-6), interleukin-18 (IL-18) and C-reactive protein (CRP). Of the antioxidants and anti-inflammatory markers, superoxide dismutase (SOD), catalase, reduced glutathione (GSH), thioredoxin, and interleukin-10 (IL-10) levels were obtained.

**Results:**

Baseline levels of oxidation markers MDA, nitric oxide and protein carbonyl groups significantly decreased after three months on paricalcitol treatment, while levels of GSH, thioredoxin, catalase and SOD activity significantly increased. After paricalcitol treatment, levels of the inflammatory markers CRP, TNF-α, IL-6 and IL-18 were significantly reduced in serum and the level of anti-inflammatory cytokine IL-10 was increased.

**Conclusions:**

In renal patients undergoing hemodialysis, paricalcitol treatment significantly reduces oxidative stress and inflammation, two well known factors leading to cardiovascular damage.

## Background

Oxidative stress is defined as the loss of balance between the generation of oxidants and the system’s antioxidant activity, in favor of the former. The production of superoxide anion, a by-product of the respiratory chain from which most free radicals derive, increases with the activity of NADPH oxidase in response to certain stimuli, including proinflammatory mediators such as complement component C5a, interleukins, tumor necrosis factor, bacterial molecules, endotoxins, etc. [1]. Maintenance of this balance is crucial in biological systems, and cells are endowed with a molecular defense system, including antioxidant enzymes such as superoxide dismutase (SOD), glutathione peroxidase and catalase, as well as vitamins (A, C, and E) and reduced glutathione (GSH). Nevertheless, some conditions or disease treatments may have an effect on this finely controlled balance, causing oxidative stress that exacerbates the patient’s condition. Such is the case of renal patients on dialysis; exposed to increased levels of oxidants due to their uremia [2,3], they have an additional load on the oxidative-inflammatory arm of this balance, caused by bioincompatible systems, adjunctive treatments (erythropoiesis-stimulating agents, intravenous iron therapy) and the antioxidant depletion caused by hemodialysis *per se*[4]. Oxidative stress in these patients leads to a state of malnutrition and accelerated cardiovascular disease [5,6].

The oxidant-antioxidant balance in renal failure has been previously studied, and dialysis treatment has been shown to increase lipid peroxidation in cell membranes and oxidation markers in the blood of treated patients [7], leading to cardiovascular dysfunction. Likewise, the characteristic apoptosis of peripheral blood leukocytes in renal patients has been associated with oxidative stress by depletion of intracellular thiol groups [8,9]. Given the body of evidence linking pathophysiological dialysis treatment with oxidative stress, which causes progressive worsening of renal patients, including cardiovascular morbidity and mortality [5,6], some authors have indicated that antioxidant therapy should be a central pillar of the preventive and curative treatment of renal disease [10,11]. Increasing evidence shows the prevalent role of vitamin D signaling pathways in redox homeostasis and cardiovascular disease prevention [12-15]. Vitamin D receptor (VDR) is present in many organs and displays pleiotropic effects; moreover, a recent clinical report indicated that intravenous calcitriol, as a VDR agonist (VDRA), reduces oxidative stress in hemodialysis patients [13].

The purpose of this study was to assess the effects of intravenous paricalcitol, a selective VDR agonist, on redox homeostasis and antioxidant systems in hemodialysis patients with secondary hyperparathyroidism (SHPT), as a possible added benefit of its use in these patients.

## Methods

This study was approved by the Clinical Research Ethics Committee of Hospital General Yagüe (Burgos, Spain). All the patients included were fully informed and gave their written consent to participate. Eligible patients were naïve to vitamin-D analogues or derivatives, had undergone regular hemodialysis three times per week for at least 12 months before the study started, and needed treatment for SHPT, presenting PTH levels ≥ 300 pg/ml, serum calcium ≥ 8 mg/dl and serum phosphorus ≤ 5.5 mg/dl. Paricalcitol dosing depended upon the levels of calcium, phosphorus and PTH. Patients presenting with PTH levels ≥ 300 pg/ml, serum calcium ≤ 9.5 mg/dl and serum phosphorus ≤ 4.5 mg/dl received 15 μg/week, distributed in three hemodialysis sessions. Those with PTH levels ≥ 300 pg/ml, serum calcium > 9.5 mg/dl and serum phosphorus > 4.5 mg/dl received 7.5 μg/week, distributed in three hemodialysis sessions. Those participants who suffered infectious or inflammatory episodes during the active phase of the study period, or who had neoplasia, were excluded from data analysis, as were those who needed transfusions or replacement of their vascular access during the study. Other reasons for exclusion were adverse effects leading to a reduction in the paricalcitol dose or treatment suspension. In the case of hyperphosphatemia, patients were treated with non-calcium binders and a controlled diet. Any concomitant treatment the patients were receiving prior to this study was maintained without variations.

Blood samples for baseline determinations were collected from each patient after inclusion and just prior to initiating paricalcitol treatment. A second sample was taken at a follow-up visit after three months of receiving paricalcitol. Extractions were performed on the day between dialysis treatments. The paricalcitol dosage for each patient was calculated according to baseline calcium, phosphorus and PTH levels. Paricalcitol doses were maintained throughout the treatment period, and were reduced only in the case of hypercalcemia or hyperphosphatemia; however they could be increased if SHPT persisted and electrolyte levels were adequate. Whole blood was used for some measurements, using heparin as anticoagulant; serum was separated following a standard protocol.

### Determination of inflammation markers in serum

The serum IL-6, IL-10, IL-18 and TNF-α levels were quantified using ELISA kits (R&D Systems, Minneapolis, USA). Serum CRP was measured using a high sensitivity turbidimetric immunoassay (hs-CRP), calcium using the calcium-o-cresolphthalein complexone method and phosphorus using the phosphomolybdate method. All were analyzed on a Roche/Hitachi Modular P analyzer ACN 210 (Roche Diagnostics, Basel, Switzerland). IDO activity was evaluated by measuring kynurenine in plasma according to Alegre *et al.*[16]. Serum PTH was quantified by electrochemiuminescence immunoassay, using a Roche Elecsys 1010/2010 analyzer (Roche Diagnostics, Basel, Switzerland). Each sample was analyzed in triplicate.

### Analysis of oxidative stress-related parameters

SOD activity was quantified with the method developed by McCord and Fridovich [17], based on the production of superoxide radicals during the conversion of xanthine to uric acid by xanthine oxidase, and the inhibition of cytochrome c reduction. One unit of SOD activity was defined as the amount of SOD that produces 50% inhibition of cytochrome c reduction. Catalase activities were assayed using the method described by Claibone [18].

Reduced GSH levels were measured in blood using the protocol of Brigelius *et al.*[19] for glutathione-S-transferase. Plasma thioredoxin was determined with an ELISA kit (TRX ELISA Kit, Redox Bioscience Inc., Japan), and malondialdehyde (MDA) was measured by HPLC [20].

Protein oxidation was assessed with an estimation of carbonyl groups formed using the protocol described by Levine *et al*. [21]. Total protein concentration was determined by the Lowry method [22], using bovine albumin as a standard. The Griess reaction [23], frequently used to indirectly measure nitric oxide content, was performed for nitrite quantification.

### Statistical analysis

All data are reported as mean ± SD. Comparison between parameter values obtained before treatment and three months after treatment initiation was performed using the paired Wilcoxon test for comparisons, since the sample size was too small for parametric tests. All statistical analyses were performed using software environment R, version 2.14.0 (Developed by international R project: http://www.r-project.org).

## Results

The study included 19 renal patients on dialysis (15 male and 4 female), between 42 and 78 years of age (mean 68 ± 13.5), with two types of vascular access: arteriovenous fistula (n= 14) or permanent internal jugular catheter (n= 5). Etiology of kidney disease in our sample was diabetes mellitus (n= 2), nephrosclerosis (n= 12), polycystic kidney disease (n= 1), and vasculitis (n= 4). All patients received treatment with EPO alfa (mean dose 5842 ± 3610 U/week, SC) as an erythropoiesis-stimulating agent; 52.6% of patients were already receiving intravenous iron (5 mg/week) two months before initiating paricalcitol treatment and iron therapy was maintained at the same dose throughout the study; 63.2% of patients were taking statins to treat dyslipidemia, and 15.8% were on angiotensin-converting enzyme (ACE) inhibitors II or angiotensin receptor blockers (ARB). The dialysis vintage had a median of 43.1 months (max. 120 - min. 12 months). Dialysis was performed for all patients using a high-flux dialyzer with a polyethersulphone membrane filter (Elisio 210H), with effective surface area 2.1 m² and dialysis solution containing 1.25-1.5 mmol/l of calcium. This dialysis protocol was maintained throughout the study. All patients had received previous treatment for SHPT: six had received calcium-chelating agents, twelve other chelating agents and one calcimimetics. Baseline oxidation and inflammation parameters were similar in all patients.

### Secondary hyperparathyroidism

The mean paricalcitol dose was 13.46 ± 3.98 μg/week and all patients included in the final analysis received paricalcitol for 12 weeks with no variations. All the patients responded to treatment, and endpoint values were compared to their baseline parameters. The dose and treatment schedule were based on the patient’s calculated requirements, as indicated in the Methods section.

Baseline PTH, calcium and phosphorus levels are shown for all the participants (Table 1). High baseline PTH levels were significantly decreased after 12 weeks of treatment with paricalcitol, while both blood calcium and phosphorus levels were increased.

**Table 1:** Effects of paricalcitol on SHPT and electrolyte levels in our sample

**N = 19**	**Paricalcitol**		P value*
	**Baseline values**	**After 12 weeks**	
**Ca,** mg/dL	8.59 ± 0.58	9.44 ± 0.71	0.00026
**P,** mg/dL	4.25 ± 0.71	4.81 ± 1.11	0.0248
**PTH,** pg/mL	441 ± 221	205 ± 172	0.00014

Ca, calcium; P, phosphorus; PTH, parathyroid hormone. Data are mean ± SD. Statistical significance assessed using Wilcoxon Signed Rank Test.

### Inflammation biomarkers

Interleukins in serum were measured in 13 patients. Paricalcitol significantly increased serum IL-10 levels. On the other hand, inflammatory cytokine levels, including IL-18, IL-6, and TNF-α were reduced after twelve weeks of treatment (Table 2). The reduction rate in serum was approximately 74%, 66%, and 53% for IL-18, IL-6, and TNF-α, respectively. However, no differences were seen in IDO activity as a result of the treatment.

**Table 2:** Inflammation markers in serum of patients before treatment initiation with paricalcitol and after 12 weeks of treatment

		**Paricalcitol**			**P value**
	**n**	**Baseline values**	**After 12 weeks**		
**CRP**, mg/L	19	21.7 ± 20.7	11.8 ± 10.3	↓	0.0076
**TNF-α**, pg/mL	13	6.15 ± 3.49	3.23 ± 1.96	↓	0.012
**IL-6**, pg/mL	13	8.96 ± 3.84	6.23 ± 1.91	↓	0.019
**IL-18**, pg/mL	13	276 ± 77	199 ± 98	↓	0.028
**IL-10**, pg/mL	13	0.39 ± 0.03	0.428 ± 0.032	↑	0.019
IDO*, μM Kyn	19	3.33 ± 1.03	3.01 ± 1.14		0.167

CRP, C-reactive protein; TNF-α, Tumor necrosis factor; IL-6, Interleukin 6; IL-18, Interleukin 18; IL-10, Interleukin 10; IDO, Indoleamine 2,3-dioxygenase, measured as kynurenine. Values are given as mean ± SD. Statistical significance assessed using Wilcoxon Signed Rank Test.

*IDO: intra-assay coefficient of variation = 2.8%, inter-assay coefficient of variation = 4.8%.

### Oxidative stress biomarkers

Compared to baseline values, increased antioxidant concentrations were observed (Table 3), including the enzymatic participants (SOD and catalase), and non-enzymatic scavengers (GSH and thioredoxin). SOD, catalase, GSH and thioredoxin levels in blood were significantly higher: 3.74, 1.35, 1.77 and 1.65-fold the baseline values. We determined the carbonyl groups and MDA levels in plasma as parameters indicative of protein and lipid oxidative damage and nitrites. As shown in Table 3, all were found to be significantly reduced after paricalcitol therapy.

**Table 3:** Enzymatic antioxidant activity and oxidation markers in the blood of dialysis patients before and after treatment with paricalcitol

**n= 19**	**Paricalcitol**			intraCV*	interCV**	**P value**
	**Baseline values**	**After 12 weeks**				
**Antioxidants**						
**SOD**, U/g Hb	734 ± 481	1776 ± 574	↑	4.8 %	3.8 %	0.00005
**CAT**, U/g Hb	2424 ± 832	3268 ± 1363	↑	2.4 %	4.03 %	0.0056
**GSH,** μmol/g Hb	1.79 ± 0.76	2.87 ± 1.24	↑	4.4 %	5.3 %	0.0002
**TRX,** ng/mL	60.2 ± 17.0	89.7 ± 22.1	↑	2.3 %	3.1 %	0.002
**Oxidation markers**						
**MDA,** μM	1.14 ± 0.18	0.96 ± 0.14	↓	3.1 %	2.8 %	0.002
**CG,** nmol/mg prot	1.78 ± 0.75	1.16 ± 0.45	↓	2.7 %	3.3 %	0.0007
**Nitrites,** μM	7.09 ± 3.42	4.33 ± 3.71	↓	3.8 %	3.4 %	0.01

SOD, Superoxide dismutase; CAT, catalase; GSH, reduced glutathione; TRX, Thioredoxin; MDA, malondialdehyde; CG, carbonyl groups; Hb, hemoglobin. Values are mean ± SD. *intra-assay coefficient of variation, **inter-assay coefficient of variation.

## Discussion

Our results suggest that treatment with paricalcitol, a VDR selective activator, has antioxidant and anti-inflammatory effects in patients on hemodialysis. This potential effect could be very beneficial for these patients, since they suffer redox imbalance due to the uremia caused by their renal condition [2]. Many treatments also induce oxidation and inflammation in hemodialysis patients, including intravenous iron therapy, erythropoiesis-stimulating agents, failed vascular access and intercurrent infectious processes [24]. Renal patients on dialysis are at risk of cardiovascular disease, due to the link of oxidative stress and inflammation with endothelial damage and immune system dysfunction and subsequent infections, perpetuating an oxidation-inflammation-infection cycle. Furthermore, the effects of the low activation of vitamin-D receptors are enhanced by the significantly reduced expression of these molecules in patients with chronic kidney disease (CKD), further contributing to endothelial dysfunction [14,15,25]. These circumstances lead inexorably to cardiovascular disease, which is a major cause of mortality in renal patients.

Therapy with vitamin D for hemodialysis patients was primarily designed to treat SHPT, regulating calcium and phosphorus absorption in the intestine and bone remodeling. This approach showed parallel benefits, as preclinical *in vitro* and observational studies found that treatment with a nonselective vitamin D activator (calcitriol) also decreased inflammatory markers [26,27] and had immunomodulatory effects [28], thus showing the potential to reduce cardiovascular damage and mortality, beyond the primarily intended control of SHPT [29]. Despite presenting excellent control of SHPT, and adding this anti-inflammatory effect, calcitriol treatment has the drawback of producing elevated serum phosphorus and calcium levels. This effect is caused by increased intestinal absorption of both electrolytes [30,31], and limits the usefulness of calcitriol in patients with severe renal disease, since the calcium and phosphorus overload cannot be adequately excreted by a failing kidney. However, the good clinical results obtained for VDRA agonists against SHTP and the need to reduce adverse effects has prompted further research for improved VDRAs. Therefore, the present study focuses on a second generation direct selective VDR agonist, 19-nor-1,25-dihydroxyvitamin D2, or paricalcitol. This selective VDRA has already shown improved effects on calcium homeostasis and SHPT treatment [31-33], controlling SHPT without increasing serum electrolyte concentrations to the levels induced by calcitriol [34,35]. In our sample, the beneficial effects on SHPT are clear, and although our patients did experience a significant increase in calcium and phosphate, the focus of our study was on other pleiotropic effects that have been reported.

The central findings of this study are the antioxidant effects of paricalcitol in a clinical setting. Redox homeostasis is a clinical concern in dialysis patients and, although hemodialysis with on-line regeneration of the ultrafiltrate has shown some improvement over conventional polysulphone dialysis in some parameters [36,37], the treatment is still related to oxidative stress, and patients undergoing hemodialysis with any of these techniques may benefit from concomitant treatments that could contribute to redox homeostasis. The anti-inflammatory/antioxidant effect of a VDRA was recently reported by Wu *et al.*[26], who carried out a study in hemodialysis patients with SHPT receiving calcitriol for 16 weeks. Paricalcitol had shown some promise regarding redox homeostasis in preclinical studies. Husain *et al.* designed an antioxidant strategy including enalapril and paricalcitol for ApoE-deficient atherosclerotic mice, since ACE inhibitors, such as enalapril, prevent formation of angiotensin II, which is known to induce free-radical generation. This combination demonstrated protective efficacy in aortic inflammatory and oxidative injury in this animal model [38]. Moreover, paricalcitol has also shown protective effects against oxidative stress in the cardiac tissue of uremic rats, through inhibition of NADPH oxidase activity [39], but there was still no clinical evidence of the anti-inflammatory/antioxidant effect of this selective VDR.

The increased levels of SOD and GSH with paricalcitol treatment found in our study follow preclinical findings with this selective VDR agonist. Since both GSH and SOD are the main contributors to cellular redox homeostasis, it is rather relevant that paricalcitol treatment seemed to increase the levels of these ROS scavengers, but we also found increased thioredoxin levels. A recent study on the effects of VDRAs on the thioredoxin pathway in endothelial cells *in vitro* indicated that paricalcitol did not modify thioredoxin expression or activity, although expression of its inhibitor, thioredoxin-interacting protein, was significantly reduced [40]. However, *in vitro* environments are rather limited and the higher levels of thioredoxin after paricalcitol treatment found in our study are consistent with the increase in other components of the antioxidant arm. The induction pathway of most of these antioxidant molecules by paricalcitol remains unknown, and further exploration of their signaling pathways could provide more information on the different mechanisms of paricalcitol and calcitriol, as suggested by their different effects.

Regarding the oxidation markers, the reduction in MDA levels by paricalcitol was also reported in an animal model, and it was postulated that the reason could be downregulation of RAS by vitamin D analogues [38]. The antioxidant effect of paricalcitol is further observed with the reduction of carbonyl groups and nitrites. The latter may be due to a previously described downregulation of eNOS and iNOS activity, which would respond both to an antioxidant and anti-inflammatory effect of VDR activation [38,41]. On the other hand, the reduced production of nitrites, MDA and carbonyl groups could also be a consequence of the increased antioxidant elements, i.e. GSH, thioredoxin and SOD, scavenging excess ROS before excessive oxidation can take place.

Modulatory effects on inflammatory and immune signaling pathways *in vitro* have been previously described, with significant reduction of proinflammatory cytokines IL-8, CRP and TNF-α and increased anti-inflammatory markers [12,42]. As previous studies have suggested, our results showed significant reduction of several inflammatory markers (CRP, TNF-α, and ILs 6 and 18) in CKD patients treated with paricalcitol, while IL-10, an anti-inflammatory cytokine, was significantly increased in the serum of our patients. No significant changes were found in IDO activity; these results have not been included in this study because IDO activity and kyneurine are increased in CKD patients and have been linked to atherosclerosis [43-45]. Permanent hemodialysis catheters are a well-known source of inflammation, and our patients had two different types of venous access, so we checked for differences in inflammatory markers between the patients with catheters and those with arteriovenous fistula, finding similar baseline and final results (data not shown), which finally allowed for a pooled analysis. Our findings are similar to those of Alborzi *et al.*[46], who observed a 20% reduction in CRP with paricalcitol. Navarro *et al.*[47] also found a significant reduction in CRP and TNF-α, while detecting higher levels of TNF-α/IL-10 and IL-6/IL-10 indices, showing an immunomodulatory effect as suggested by our own results. Our findings in this group are consistent with previous reports on clinical data and experimental studies in rats [12,42,48].

The results of this clinical study, conducted on 19 patients with hemodialysis, suggest another important effect of paricalcitol, regarding renal disease complications. This is the first report on the antioxidant effects of a VDR agonist, namely the selective agonist paricalcitol, in a clinical study involving renal patients. One limitation of this study is the absence of a placebo arm, as our study was performed in the setting of routine clinical practice and comparison with a control group was not possible. However, comparison of final values, with baseline analyses of the same patients acting as control, showed significant changes after paricalcitrol treatment; these very significant changes in so many different parameters are unlikely to be observed in an untreated control group as a result of spontaneous evolution. Moreover, these improvements in redox homeostasis could be observed after a short treatment period of three months indicating that, although the study period was very short, three months was also enough to observe significant changes. Since this study was conducted in a clinical setting and the patients were receiving concomitant medication, an association between oxidative stress/inflammation and other medications commonly adopted in dialysis patients, such as ACE inhibitors, ARBs or chelating agents could be suspected [49-51]. However, the participants’ concomitant medication was started before the study and maintained without modifications during the study, so the possible effects of these drugs were constant. Although the laboratory results of the three patients who were receiving ACE inhibitors were well within the range of values obtained for the rest of the patients, we performed a sensitivity analysis excluding these patients to determine any influence that this medication might have in our results, but the level of statistical significance did not change substantially (data not shown). The main limitations are the small sample size and the lack of a control group or randomization, with possible selection bias, since the setting and circumstances in which it was performed did not allow improvements in this aspect.

## Conclusions

Hemodialysis patients receiving this selective VDR agonist could benefit not only from SHPT control but also from anti-inflammatory and antioxidant effects that could improve their situation in terms of cardiovascular protection. We consider the results of this study promising, as improvements were seen in a very short follow-up period, although a larger trial comparing treatment with paricalcitol, other VDR agonists and placebo could provide conclusive data on the topic.

## Competing interests

Dr de Francisco is consultant to Amgen Fresenius and has received speaker′s honoraria from Abbott, Roche, Rubió, Shire, and Genzyme on occasion. Dr. Izquierdo has received speaker’s honoraria form Abbott on occasion. The other authors have no conflicts of interests regarding the contents of this manuscript, and the study has not received any funds from Industry.

## Authors' contributions

MJI participated in the design of the study, performed patient selection, managed the data and drafted the manuscript. MC and PM carried out the biochemical analyses. AF and MA conceived the study, participated in its design and collaborated in the manuscript. JA participated in patient selection and PA in the design and coordination. All authors read and approved the final manuscript.

## Pre-publication history

The pre-publication history for this paper can be accessed here:

http://www.biomedcentral.com/1471-2369/13/159/prepub
